# Novel HBsAg mutations correlate with hepatocellular carcinoma, hamper HBsAg secretion and promote cell proliferation *in vitro*

**DOI:** 10.18632/oncotarget.14944

**Published:** 2017-02-01

**Authors:** Romina Salpini, Matteo Surdo, Nadia Warner, Maria Francesca Cortese, Danny Colledge, Sally Soppe, Maria Concetta Bellocchi, Daniele Armenia, Luca Carioti, Fabio Continenza, Domenico Di Carlo, Patrizia Saccomandi, Carmen Mirabelli, Michela Pollicita, Roberta Longo, Sara Romano, Giuseppina Cappiello, Alberto Spanò, Pascale Trimoulet, Herve Fleury, Jacopo Vecchiet, Nerio Iapadre, Angelo Barlattani, Ada Bertoli, Terenzio Mari, Caterina Pasquazzi, Gabriele Missale, Cesare Sarrecchia, Elisa Orecchini, Alessandro Michienzi, Massimo Andreoni, Simona Francioso, Mario Angelico, Jens Verheyen, Francesca Ceccherini-Silberstein, Stephen Locarnini, Carlo Federico Perno, Valentina Svicher

**Affiliations:** ^1^ Department of Experimental Medicine and Surgery, University of Rome “Tor Vergata” Rome, Italy; ^2^ Research and Molecular Development, Victorian Infectious Diseases Reference Laboratory, North Melbourne, Victoria, Australia; ^3^ Laboratory of Monitoring Antiviral Drugs, National Institute for Infectious Diseases (INMI) “Lazzaro Spallanzani” Rome, Italy; ^4^ Institut Pasteur, Unité de Biologie des Virus Entériques, Paris, France; ^5^ Unit of Microbiology, “S. Pertini Hospital”, Rome, Italy; ^6^ Laboratoire de Microbiologie Fondamentale et Pathogénicité, Hôpital Pellegrin Tripode, Bordeaux, France; ^7^ Department of Medicine and Aging Sciences, “SS Annunziata” Hospital, Chieti, Italy; ^8^ Infectious Diseases Unit, “S Salvatore” Hospital, L'Aquila, Italy; ^9^ Hepatology Unit, “S Giacomo” Hospital, Rome, Italy; ^10^ Hepatology Unit, “Regina Margherita” Hospital, Rome, Italy; ^11^ Hepato-Infectivology Unit, “S Andrea” Hospital, Rome, Italy; ^12^ Hospital of Parma, Parma, Italy; ^13^ Tor Vergata University Hospital, Infectious Diseases Unit, Rome, Italy; ^14^ Department of Biomedicine and Prevention, University of Rome “Tor Vergata” Rome, Italy; ^15^ Tor Vergata University Hospital, Hepatology Unit, Rome, Italy; ^16^ Institute of Virology, University Hospital, University of Duisburg-Essen, Essen, Germany

**Keywords:** hepatitis B, hepatocellular carcinoma, hepatitis B surface antigen, HBsAg mutations, cell proliferation

## Abstract

**Background:**

An impaired HBsAg-secretion can increase HBV oncogenic-properties. Here, we investigate genetic-determinants in HBsAg correlated with HBV-induced hepatocellular carcinoma (HCC), and their impact on HBsAg-secretion and cell-proliferation.

**Methods:**

This study included 128 chronically HBV-infected patients: 23 with HCC (73.9% D; 26.1% A HBV-genotype), and 105 without cirrhosis/HCC (72.4% D, 27.6% A) as reference-group. The impact of mutations on HBsAg-secretion was assessed by measuring the ratio [secreted/intracellular HBsAg] until day 5 post-transfection. The impact of mutations on cell-cycle advancement was assessed by flow-cytometry.

**Results:**

Two HBsAg mutations significantly correlated with HCC: P203Q (17.4% [4/23] in HCC vs 1.0% [1/105] in non-HCC, P=0.004); S210R (34.8% [8/23] in HCC vs 3.8% [4/105] in non-HCC, P <0.001); P203Q+S210R (17.4% [4/23] in HCC vs 0% [0/110] in non-HCC, P=0.001). Both mutations reside in trans-membrane C-terminal domain critical for HBsAg-secretion. In *in-vitro* experiments, P203Q, S210R and P203Q+S210R significantly reduced the ratio [secreted/intracellular HBsAg] compared to wt at each time-point analysed (P <0.05), supporting an impaired HBsAg-secretion. Furthermore, P203Q and P203Q+S210R increased the percentage of cells in S-phase compared to wt, indicating cell-cycle progression (P203Q:26±13%; P203Q+S210R:29±14%; wt:18%±9, P <0.01. Additionally, S210R increased the percentage of cells in G2/M-phase (26±8% for wt versus 33±6% for S210R, P <0.001).

**Conclusions:**

Specific mutations in HBsAg C-terminus significantly correlate with HBV-induced HCC. They hamper HBsAg-secretion and are associated with increased cellular proliferation, supporting their involvement in HCC-development. The identification of viral genetic markers associated with HCC is critical to identify patients at higher HCC-risk that may deserve intensive liver monitoring, and/or early anti-HBV therapy.

## INTRODUCTION

Hepatocellular carcinoma (HCC) is one of the most frequent solid tumors and the third cause of cancer death worldwide [1, 2]. This cancer is rapidly fatal in the majority of patients with advanced liver disease. Recent estimates attribute to hepatitis B virus (HBV) over 50% of HCC cases worldwide. The lifetime risk of developing HCC is 10- to 100- fold greater for chronic HBV carriers than non-infected individuals. In addition, individuals with clinically resolved HBV infection have a risk to develop HCC ≈ 3-fold greater than uninfected populations. In contrast with HCV, a substantial number of HBV-infected patients develops HCC without any sign of liver damage [3], highlighting the existence of intrinsic HBV-mediated pro-oncogenic mechanisms [1, 4–9].

At this moment, there is paucity of diagnostic tools able to predict the development of HCC; still today, the prevention is based essentially on ultrasound-driven early diagnosis of cancer development. Despite this effort, the prognosis of HCC remains poor [10]. This strongly highlights the need to identify patients at higher risk to develop HCC in order to set up a more intensive liver monitoring and/or an early treatment [11, 12].

Viral genetic variability plays an important role in potentiating HBV pro-oncogenic properties. Several studies have correlated the presence of mutations in HBV genomic region known as basal core promoter and HBx with an increased risk to develop HCC [13–16]. A growing number of studies is also pointing out the role of HBV surface antigen in mechanisms underlying HBV-related carcinogenesis [5].

The S-gene is composed by pre-S1, pre-S2, and S region, coding for the three forms of HBV surface glycoproteins: the small-sized HBV surface antigen (S-HBsAg), the medium-sized (M-HBsAg, pre-S2+S) and large-sized surface antigen (L-HBsAg, pre-S1+pre-S2+S). HBV surface proteins (L-HBsAg, M-HBsAg, and S-HBsAg) are synthesized in the endoplasmic reticulum where they rapidly undergo dimer and multimer formation via extensive disulphide bonding. This results in budding into the endoplasmic reticulum as either spherical or filamentous empty subviral particles (mainly composed by S-HBsAg), or as virions [17]. Stop codons in S-HBsAg as well as deletions in L-HBsAg and M-HBsAg have been associated with the production of truncated or structurally-modified surface antigens, that own a dominant negative secretion defect, and accumulate inside the hepatocytes [8, 18, 19]. The intracellular accumulation of unfolded or misfolded HBV surface antigens exerts cytotoxic effects, activating intracellular signaling pathways associated with oxidative stress, thus promoting cell transformation [7, 8]. Beyond deletions and stop-codons, paucity of information is available on point mutations in surface antigens correlated with HCC.

Among the different S-HBsAg domains, the C-terminus (from amino acid [aa] 179 to 226) is hydrophobic and is assumed to be inserted in the endoplasmic reticulum membrane [20]. This domain is involved in mediating the transit of the surface glycoproteins across the endoplasmic reticulum [21]. Mutations in this domain can result in a stable, glycosylated, but non-secreted chain, thus affecting the biogenesis and secretion of subviral particles [21]. Thus, it could be hypothesized that mutations in this domain could promote an intra-cellular accumulation of S-HBsAg and in turn contribute to promote cell proliferation [1, 5, 22]. So far, no studies have addressed this issue.

Based on this rationale, this study is aimed at evaluating i) the correlation of mutations in the C-terminus S-HBsAg domain with HBV-induced HCC, ii) the impact of these mutations on HBsAg secretion, iii) the impact of these mutations on cell-cycle progression. The identification of these viral markers is critical in order to identify patients at higher risk to develop this deadly disease that need an early treatment and/or a more intensive liver monitoring. This represents an unmet medical need, fully answering to the issue “assess host genetic and viral markers to determine prognosis and optimise patients' management” raised by European HBV Guidelines promoted by the European Association for the Study of the Liver (EASL) (http://www.easl.eu/research/our-contributions/clinical-practice-guidelines).

## RESULTS

### Patients' characteristics

This study included 23 patients with a diagnosis of HBV-induced HCC (Table 1). Most patients were male (91.7%), with a median (interquartile range [IQR]) age of 63 (53-70) years (Table 1). At HCC diagnosis, median (IQR) HBV-DNA was 4.0 (2.1-6.5) log IU/ml and median (IQR) ALT and AST was 82 (28-112) and 50 (33-139) IU/L, respectively (Table 1). Notably, 47.8% of patients received HCC diagnosis in a condition of low-grade liver disease (Child-Pugh class A), and 34.8% developed HCC in absence of liver cirrhosis (Table 1). At HCC diagnosis, most patients showed an elevated level of alpha-fetoprotein (AFP) (median [IQR] serum AFP: 519[6.2-13,012] ng/ml) (Table 1). In most patients (60.9%), liver imaging showed the presence of a single-nodule HCC, with a median (IQR) size of 5 (2.5-7.6) cm, while in 30.4% of patients a multi-focal HCC was diagnosed (Table 1). Among 17 patients suitable for HCC treatment, trans-catheter arterial chemoembolisation (TACE) was the most used strategy for HCC treatment (in 8/17 [47.1%]) while in 4/17 (23.5%) the progression of HCC required to be treated with a surgical resection (Table 1). At HCC diagnosis, 60.9% of patients were treated with a nucleos(t)ide analogues (NUCs) (Table 1).

**Table 1:** Characteristics of the study population

Patients’ characteristics	HCC patients(N=23)	Control patients(N=105)
Male, N(%)	21 (91.7)	80 (76.2)
Italian Nationality, N(%)	16 (69.6)	89 (84.8)
Median Age [Years] (IQR)	63 (53-70)	51 (41-65)
HBeAg negative, N(%)^a^	14 (77.8)	62 (78.5)
Median HBV-DNA [log IU/ml] (IQR)	4.0 (2.1-6.5)	4.1 (3.1-5.4)
Median ALT [IU/L] (IQR)	82 (28-112)	49 (29-90)
Median AST [IU/L] (IQR)	50 (33-139)	35 (23-58)
**HBV Genotype**		
D, N(%)	17 (73.9)	76 (72.4)
A, N(%)	6 (26.1)	29 (27.6)
**Anti-HBV Therapy**		
None, N(%)	9 (39.1)	45 (42.9)
LMV, N(%)	6 (26.1)	30 (28.6)
ETV, N(%)	3 (13.0)	3 (2.9)
TDF, N(%)	3 (13.0)	15 (14.3)
ADV, N(%)	1 (4.4)	9 (8.6)
LMV+ADV, N(%)	1 (4.4)	3 (2.9)
**HCC characteristics**		
Single Nodule HCC, N(%)	14 (60.9)	
Multifocal-HCC, N(%)	7 (30.4)	
Unknown, N(%)	2 (8.7)	
**Tumor size**		
Median size of each HCC nodule [cm] (IQR)	5 (2.5-7.6)	
**Median alfa-feto protein at HCC diagnosis (IQR), ng/ml**	519 (6.2-13,012)	
**Portal Invasion**		
Yes, N(%)	5 (21.7)	
No, N(%)	14 (60.9)	
Unknown, N(%)	4 (17.4)	
**Capsular Formation**		
Yes, N(%)	7 (30.4)	
No, N(%)	7 (30.4)	
Unknown, N(%)	9 (39.2)	
**HCC treatment**		
TACE, N(%)	8 (34.8)	
Surgical resection, N(%)	4 (17.4)	
Thermoablation, N(%)	2 (8.7)	
No, N(%)	3 (13.0)	
Unknown, N(%)	6 (26.1)	
**Liver status**		
Diagnosis of cirrhosis, N(%)	15 (65.2)	
**Child-Pugh Classification**		
A, N(%)	11 (47.8)	
B, N(%)	6 (26.1)	
C, N(%)	2 (8.7)	
Unknown, N(%)	4 (17.4)	

a The percentages were calculated on 18 HCC and 79 control patients with an available HBeAg status.

Abbreviations: HCC, Hepatocellular carcinoma; HBeAg, Hepatitis B “e” antigen; ALT, Alanine Aminotransferase; AST, aspartare aminotrasferase; TACE, Transcatheter arterial Chemoembolisation; LMV, lamivudine; ETV, entecavir; TDF, tenofovir disoproxil fumarate; ADV, adefovir.

### HBsAg mutations associated with HBV-related HCC

The correlation of mutations in the C-terminus region of S-HBsAg was investigated. A group of 105 patients with chronic hepatitis B and no clinical evidence of cirrhosis and hepatocellular carcinoma was used as reference. These patients had comparable virological parameters in terms of serum HBV-DNA, percentage of HBeAg negativity, HBV genotype distribution and experience of anti-HBV therapy (Table 1).

**Figure 1:**
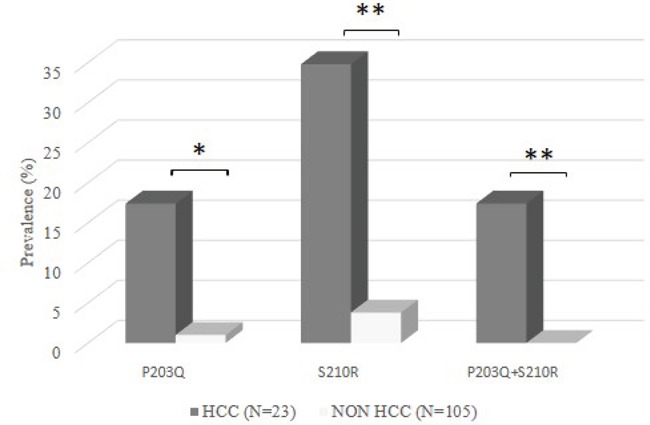
The histogram reports the prevalence of sP203Q, sS210R, and sP203Q+sS210R in 23 HBV-induced HCC patients and in 105 chronically HBV-infected patients (CHB patients) (used as reference group) Statistically significant differences were assessed by Fisher Exact Test. * P <0.05, ** P <0.01.

Two specific mutations (sP203Q and sS210R) were significantly correlated with the presence of HCC (Figure 1). sP203Q occurred in 17.4% (4/23) of HCC-patients versus 1.0% (1/105) of patients without HCC (P=0.004) (Figure 1). Similarly, sS210R was found in 34.8% (8/23) of HCC-patients and in 3.8% (4/105) of patients without HCC (P <0.001) (Figure 1). In particular, the double mutant sP203Q+sS210R was detected exclusively in HCC-patients (17.4% [4/23] versus 0% [0/105], P=0.001) (Figure 1). sP203Q occurred as a mixture in 2/4 HCC patients while sS210R occurred as a mixture only in 1 patient. By analysing the overlapping between the HBsAg and RT genes, sP203Q did not result in any amino acid substitution in RT, while sS210R corresponded to rtS219A. This mutation is localized in an RT region (whose function has not been defined yet) between the RT domains C (aa: 200-210) and D (aa: 230-241). The percentage of patients with sS210R/rtS219A was comparable between drug naïve HCC-patients (33.3%, 3/9) and drug-treated HCC-patients (35.7%, 5/14), thus excluding the impact of antiviral therapy on the selection of this mutation.

Multivariate analysis confirmed that the presence of either sP203Q or sS210R was independently associated with a higher probability of developing HCC (adjusted Odd-Ratio [95% C.I.]: 12.0 [1.6-78.8], P=0.02), after correction for patients' age, gender, HBV genotype, serum HBV-DNA, ALT and AST (Table 2).

**Table 2:** Multivariate analysis: factors associated with HCC

Variables^a^	Univariate analysis		Multivariate analysis	
	crude OR [95% CI]	p-value	adjusted OR [95% CI]	p-value
**Gender (Female vs. Male^b^)**	0.30 (0.07-1.39)	0.125	0.27 (0.04-1.96)	0.197
**Age (for 1 year increase)**	**1.06 (1.02-1.10)**	**0.004**	**1.06 (1.01-1.12)**	**0.019**
**Year (for 1 year increase)**	0.96 (0.84-1.09)	0.499	0.99 (0.83-1.18)	0.902
**HBV-DNA (log_10_)**	0.98 (0.77-1.25)	0.857	0.91 (0.66-1.26)	0.588
**ALT**	0.98 (0.97-1.01)	0.993	0.99 (0.98-1.01)	0.212
**AST**	1.01 (0.98-1.02)	0.422	1.01 (0.99-1.03)	0.188
**Genotype (A vs D^b^)**	0.92 (0.33-2.58)	0.881	0.85 (0.19-3.73)	0.826
**At least one mutation (P203Q+/−S210R)**	**13.47 (3.61-50.27)**	**<0.0001**	**6.45 (1.37-30.43)**	**0.019**

a The logistic regression analysis was performed on 128 chronically HBV-infected patients with or without HCC. The following variables were considered: gender, year of sample collection, age, HBV-DNA log10, ALT, AST, HBV genotype, at least one mutation associated with HCC.

b Reference group

Furthermore, multifocal HCC was found to occur more frequently in patients with either P203Q or S210R (4/8 [50%]) than in patients without these mutations (3/15 [20%]). In line with this datum, the level of alpha-fetoprotein (AFP) at HCC diagnosis was higher in patients with either P203Q or S210R than in patients without these mutations (median [IQR] AFP: 6500 [212–18250] ng/mL vs 58 (7-2139) ng/mL). No other mutations in C-terminus of HBsAg resulted significantly correlated with HCC.

Previous studies have correlated the presence of deletions in the preS1 and preS2 regions in S gene with an increased HBV oncogenic potential [5]. Thus, the presence of pre-S1 and pre-S2 deletions was investigated, as well as their co-occurrence with sP203Q or sS210R. Pre-S1 and Pre-S2 deletions were found in 3/23 (13.0%) HCC-patients and in 6/105 (5.7%) controls (P=0.2). No deletions were found with sP203Q or sS210R in both HCC and control group. This suggests that sP203Q/sS210R in HBsAg C-terminus and deletions in pre-S1 and pre-S2 lie on divergent evolutionary pathways contributing to HCC development.

### Data from UDPS: intra-patient prevalence of mutations associated with HCC

**Figure 2:**
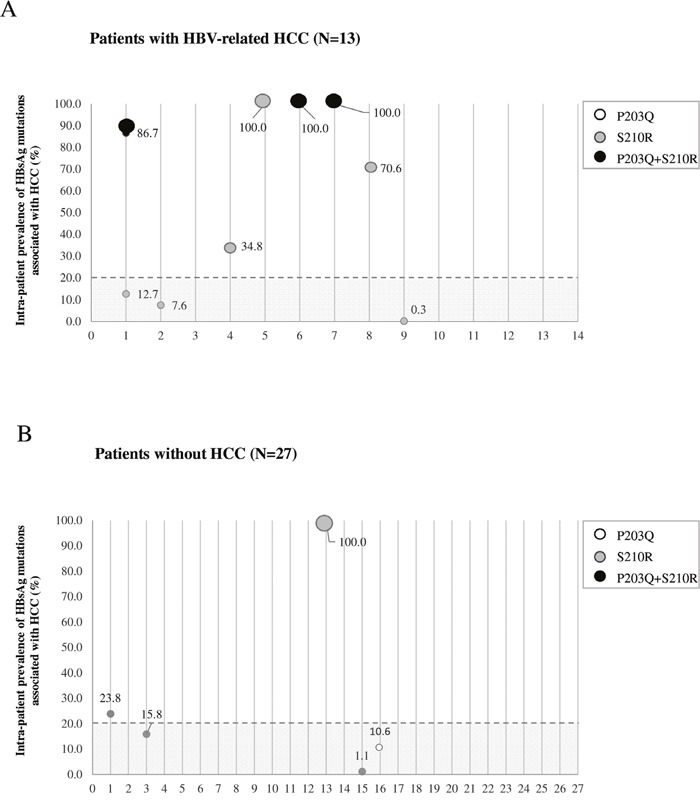
The graph reports the intra-patient prevalence of sP203Q (white dots), sS210R (grey dots), sP203Q+sS210R (black dots) for each patient in the HCC group (N=13) **A**. and in the group of chronically-infected patients used as reference (N=27) **B**. Intra-patient prevalence was expressed as % of reads with the specific mutation. The grey area includes mutations with an intra-patient prevalence <20%, not detected by standard population-based sequencing, and thus defined as minority species.

UDPS analysis was performed in 13 HCC patients (6/13 carrying sP203Q or sS210R by population-based sequencing) and 27 reference patients (2/27 carrying sP203Q or SS210R by population-based sequencing) (Figure 2). In HCC patients, sP203Q and/or sS210R occurred with a median (IQR) intrapatient prevalence of 70.6% (12.7%-100%). sP203Q never occurred as single mutation but only in association with sS210R, with an intrapatient ranging from 86.7% to 100% (Figure 2A). Conversely, in chronically infected control patients, the median [IQR] intra-patient prevalence of these HBsAg mutations, found associated with HCC, was remarkably lower (15.8% [10.6%-23.8%]) (Figure 2B).

### Impact of sP203Q, sS210R and sP203Q+sS210R mutations on the extracellular on intracellular HBsAg ratio

The next step of this study was to investigate the impact of sP203Q, sS210R and sP203Q+sS210R on HBsAg release. For this purpose, the ratio of extracellular on intracellular HBsAg was measured for the wild-type virus and each mutant. This ratio was defined as HBsAg secretion factor.

**Figure 3:**
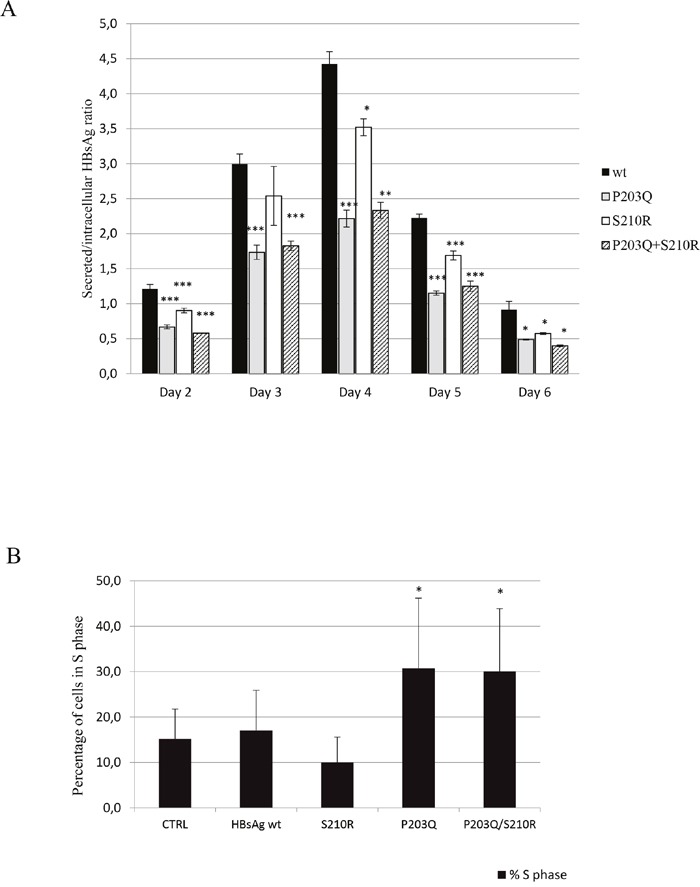
**A**. The histogram reports HBsAg secretion factor for the wild-type virus and for mutants carrying P203Q, S210R and P203Q+S210R. Secretion factor is defined as the ratio of extracellular to intracellular HBsAg amount determined by the Alexxis assay. Average of 3 experiments +/− SEM are shown. Statistically significant differences were assessed by 2-tailed unpaired T-test. *P <0.05 **P <0.01, ***P <0.005. **B**. The histogram reports the percentage of cells in S-phase of cell cycle for wild-type and mutants at 7 days post transfection determined by FACS analysis, selecting GFP+ HepG2 cells. Statistical analysis was assessed by Chi-Square Test based on 2×2 contingency table. *P <0.001

All three mutants carrying sP203Q, sS210R, and sP203Q+sS210R determined a reduction of HBsAg secretion factor compared to wild-type at each time-point analyzed (P <0.05) (Figure 3A). In particular, at day 4 of culture (characterized by the peak in HBsAg production), HBsAg secretion factor decreased from 4.42±0.3 for wild-type to 2.22±0.2 for P203Q (P <0.001), to 3.52±0.2 for S210R (P <0.05), and to 2.33±0.2 for P203Q+S210R (P <0.01) (Figure 3A). These results reflects the ability of sP203Q, sS210R and sP203Q+sS210R to negatively affect HBsAg release.

### Impact of sP203Q, sS210R and sP203Q+sS210R mutations on cell proliferation

The impact of sP203Q, sS210R and sP203Q+sS210R on cell proliferation was then evaluated by flow cytometry (DNA propidium iodide-staining) on transfected GFP+ cells at day 7 post transfection (Figure 3B). In this analysis, sP203Q and sP203Q+sS210R significantly correlated with an increased percentage of cells in the S phase of cellular cycle, thus supporting an induction of cell cycle progression (Figure 3B). In particular, the percentage of cells in the S phase at 7 days post transfection increased from 18%±9 for wild-type up to 26±13% for P203Q and 29±14% for P203Q+S210R (P <0.001 for both) (Figure 3B). Conversely, sS210R significantly correlated with an increased percentage of cells in the G2/M phase of cellular cycle (26±8% for wild type versus 33±6% for sS210R, P <0.001), thus supporting an induction of cell cycle progression and cell division (data not shown). Notably, cells transfected with HBsAg plasmid carrying the mutation sS210R showed increased intracellular levels of cyclin A, compared to those transfected with wild type HBsAg (fold compared to mock = 2.1±0.08 versus 1.7±0.04 for wt). No differences in intracellular cyclin levels were observed in hepatoma cells transfected with plasmid carrying P203Q and P203Q+S210R HBsAg mutations compared to wt (fold compared to mock = 1.8±0.2 and 1.8±0.1 versus 1.7±0.04 for wt, respectively). Therefore, the sS210R mutants within C-terminal HBsAg might contribute to cell cycle progression by modulating the expression of cyclin A.

## DISCUSSION

This study shows that sP203Q and sS210R mutations, in HBsAg C-terminal domain (aa 179 to 226), significantly correlate with the presence of HBV-induced HCC. By multivariable analysis, the presence of at least 1 of these mutations is an independent factor significantly correlated with HCC, after correction for both viral and patients' parameters. In addition, the prevalence of these mutations is comparable between drug naïve and drug treated HCC-patients, excluding the role of antiviral treatment on their selection. In *in-vitro* experiments, these mutations determine a reduction in the extracellular/intracellular HBsAg ratio, supporting their ability to hamper HBsAg secretion. In addition, sP203Q correlates with an increased percentage of cells in S phase, while sS210R with an increased percentage of cells in G2/M phase of cell cycle compared to wild-type, highlighting their ability to promote cell-cycle advancement and/or cell proliferation. To support this finding, the presence of these mutations is also correlated with increased levels of AFP *in vivo* (a well known marker of hepatocellular proliferation). Overall data support the involvement of these two mutations in mechanisms underlying HBV oncogenic properties. In addition, in a patient carrying the mutation sS210R, a pre-HCC HBV sequence was available and confirmed the presence of this mutation already 1 year before the diagnosis of HCC, further supporting the role of this mutation in promoted HBV-mediated hepatocarcinogenesis.

The identification of viral genetic markers associated with HCC is critical to identify patients at higher HCC-risk that may deserve intensive liver monitoring, and/or early anti-HBV therapy. At the same time, this may allow to reduce the elevated costs associated with the clinical management of patients with HCC. This represents an unmet medical need, fully answering to the issue “assess host genetic and viral markers to determine prognosis and optimise patients' management” raised by European HBV Guidelines promoted by the European Association for the Study of the Liver (EASL) (http://www.easl.eu/research/our-contributions/clinical-practice-guidelines).

Previous studies have investigated the role of stop-codons in S-HBsAg in mechanisms underlying HBV-induced tumorigenesis [2, 19, 25]. They showed that stop-codons (at S-HBsAg position 172, and 182) can determine the production of truncated S-HBsAg that can be retained inside the hepatocytes, thus promoting metabolic alterations associated with neoplastic transformation of the hepatocytes [1, 2, 19, 25].

This is the first study showing the involvement of specific point mutations, within S-HBsAg C-terminus, in HCC development. During HBV life cycle, HBsAg C-terminus is embedded in the membrane of the endoplasmic reticulum and plays an important role in the biosynthesis and release of sub-viral particles [21]. In particular, Bruss and Ganem found that deletions in the S-HBsAg C-terminus hamper the budding or intracellular transport of sub-viral particles [21]. In our study, the presence of sP203Q and sS210R in HBsAg C-terminus determines a decrease of the extracellular on intracellular HBsAg ratio, reflecting the ability of these mutations to affect HBsAg secretion. Notably, sP203Q and sS210R introduce a negatively and positively charged amino acid, respectively. It is conceivable that the acquisition of charged amino acids can obstacle the proper folding of HBsAg C-terminus in the endoplasmic reticulum membrane, thus impairing HBsAg release and resulting in its accumulation in specific intracellular compartments (presumably represented by endoplasmic reticulum/Golgi apparatus). In line with this hypothesis, a previous study showed that the sS204R mutation in S-HBsAg C-terminus (along with G145R) exhibited a viral secretion defect compared to the wild-type [26].

HBsAg retention within hepatocytes is a mechanism known to promote the initiation of HBV-driven carcinogenesis by activation of endoplasmic reticulum-stress-signaling dependent pathways [1, 27–29]. In line with this concept, we found that the presence of sP203Q and sS210R increases the % of cells in S- and G2/M phase of cell cycle, respectively, suggesting their contribution to the advancement of cell cycle that may pose the basis for an increased cell proliferation. Again, no studies have previously addressed this issue.

The increased amount of cyclin A observed *in vitro* for the mutant sS210R corroborates the impact of this mutation on cell cycle progression. Indeed, cyclin A plays an important role in the S and G2/M phases checkpoint of the cell cycle [30]. Several studies [31, 32] have demonstrated that an upregulation of cyclin A causes an impaired control of cell cycle progression, that contribute to the oncogenic transformation of hepatocytes. In literature, the overexpression of cyclin A was reported to link malignant features not only in hepatocellular carcinoma but also in prostate, colorectal and renal cell cancers [33–35].

Previous studies have correlated the presence of deletions in the preS1 and preS2 regions in S gene with an increased HBV oncogenic potential [5, 36]. In our study, no deletions were found in HCC-patients with sP203Q or sS210R. This may suggest that the co-presence of sP203Q/sS210R in HBsAg C-terminus and deletions in pre-S1 and pre-S2 may be lethal for HBV replication. At the same time, this may also suggest that sP203Q or sS210R and deletions in pre-S1/pre-S2 may lie on divergent evolutionary pathways contributing to HCC development.

This study was conducted in patients infected with HBV genotype D and A. Therefore, these results should be seen in the context of genotype D/A, and additional studies are required to evaluate whether these mutational pathways lead to similar conclusions also in other HBV genotypes, whose oncogenic potential has been well demonstrated.

In conclusions, specific mutations, residing in C-terminal HBsAg domain, are highly correlated with HBV-induced HCC *in vivo*. They affect HBsAg secretion and stimulate cell proliferation *in vitro*, supporting their involvement the initiation of HBV-driven carcinogenesis. The identification of viral genetic markers associated with HCC is critical to identify patients at higher HCC-risk that may deserve intensive liver monitoring, and/or early anti-HBV therapy.

## MATERIALS AND METHODS

### Study population

This study included 128 chronically HBV-infected patients (93 genotype D and 35 genotype A): 23 consecutive patients with HBV-related HCC and 105 randomly-selected patients with no clinical evidence of cirrhosis and hepatocellular carcinoma (used as reference group), followed in different clinical centers in Italy and in France from 2004 to 2014. The distribution of HBV genotype D and A in HCC- and non-HCC patients was comparable (73.9% and 26.1% in patients with HCC versus 72.4% and 27.6% in patients without HCC, respectively), minimizing the possible influence of HBV genotypes in the analysis of the two population groups.

HBV-related HCC cases were included in the study according to a radiological or histological diagnosis of a hypervascular liver tumor mass (> 1 liver cancer nodule) in patients with a previously diagnosed HBV-chronic infection. Histological staging of liver tumors was assessed according to the WHO grading system. Chronically HBV-infected patients had no clinical evidence of cirrhosis and hepatocellular carcinoma. No patients (0/128) had a co-infection with human immunodeficiency virus (HIV), hepatitis delta virus (HDV) and hepatitis C virus (HCV).

Ethic approval was deemed unnecessary because, under Italian law, biomedical research is subjected to previous approval by ethics committees only in the hypothesis of clinical trials on medicinal products for clinical use (art. 6 and art. 9, leg. decree 211/2003). The research was conducted on viral DNA samples (used for clinical routine), and data previously anonymized, according to the requirements set by Italian Data Protection Code (leg. decree 196/2003). Thus, all the plasma samples used in this study were archival, and not collected for this study. Patient data were stored anonymously in a database including genotypic, demographic, biochemical and virologic parameters.

### Population-based sequencing of the full-length S-gene (pre-S1, pre-S2, and S)

The population-based sequencing of the full-length S-gene was performed on plasma samples, following a home-made protocol, as previously described [23, 24]. Details on such protocol are reported in Supplementary-text I. The population-based sequencing of S gene was performed on the plasma samples closest to the diagnosis of HCC (never exceeding 12 months after HCC diagnosis).

Sequences having a mixture of wild-type and mutant residues at single positions were considered to have the mutant(s) at that position.

### HBV ultra-deep pyrosequencing (UDPS)

The extent of genetic heterogeneity in S-gene was also investigated by UDPS (Roche 454-Junior), according to the methodology described in Salpini et al., Hepatology 2015 [24]. Details on such protocol are reported in Supplementary-text II.

UDPS was performed for 13 out of the 23 HCC cases and for 27 out of the 105 reference patients, included in the study, on the basis of the availability of plasma samples. In HCC-patients, no statistically significant differences in clinical and demographic characteristics, as well as in the prevalence of mutations associated with HCC were found after stratification for UDPS availability (Supplementary Table S1). Similarly, in the reference group, all the demographic and clinical characteristics were comparable after stratification for UDPS availability (Supplementary Table S1).

### Association of mutations with HCC

S-HBsAg sequences obtained by population-based sequencing were used to assess the association of specific mutations in C-terminus with HBV-related HCC. Mutations were defined according to the reference sequence of each specific HBV genotype (reference sequences: X65259.1 and X02763.1 for D and A genotypes, respectively). In particular, the prevalence of each mutation was calculated in the group of HCC-patients and in the group of chronically HBV-infected patients. Fisher exact test was performed to verify whether the differences in frequency between the two groups of patients were statistically significant.

In order to support their correlation with HBV-related HCC, a logistic regression analysis was performed using the statistical open source environment R (version 3.1.1). The following variables were considered: gender, year of sample collection, age, nationality, HBV-DNA log10, ALT, AST, HBV genotype, at least one mutation associated with HCC. After stepwise elimination for optimized Akaike information criterion (AIC), only variables showing a p-value < 0.200 in univariate analysis were included in the multivariate analysis.

The sequences obtained by UDPS were used to define the intra-patient prevalence of mutations associated with HBV-related HCC.

### Cells

Huh7 and HepG2 cells were cultured in T25 flask (Costar) at a density of 5×10^5^ cells/ml in DMEM (High glucose - SIGMA) supplemented with 100 U/ml penicillin + 100μg/ml streptomycin, 2 mM GlutaMAX, and 10% fetal bovine serum (FBS) heat inactivated. Cells were grown in a 37°C humidified atmosphere containing 5% CO_2_.

### Plasmids, transfection and HBsAg quantification

S-HBsAg mutations associated with HCC were introduced into a 1.3x genome-length HBV genotype D, HBeAg negative clone using primers as previously described [2]. Primers used for mutagenesis were reported in Supplementary-text III.

Clones encoding the mutations associated with HCC, as well as wild-type (wt) HBV, were transfected into Huh7 cells by using Fugene 6 (Promega) according to manufacturer. Both cells lysates and cultures supernatants were harvested in triplicate daily, from day 2 to day 6 post-transfection, and HBsAg quantified using Alexxis qHBsAg assay. HBsAg from supernatants represents 24 hours of accumulation.

The plasmid pCMV-HBV (containing an HBV genotype-D genome, serotype *ayw*) was used as substrate to amplify the full-length S-gene by using the Expand High Fidelity PCR System (Roche) and the following primers: F- 5′-GGGGGG AGATCT ATGGGGCAGAATCTTTCCAC-3′ and R- 5′-GGGGGG GTCGAC TTAAATGTATACCCAAAGAC-3′. The resulting PCR product was digested with BglII and SalI restriction enzymes and subcloned in the corresponding sites of the pIRES2-EGFP expression vector (Clontech) generating the pIRES/ HBsAg plasmid. This plasmid encodes the three forms of HBsAg (L-HBsAg, M-HBsAg, S-HBsAg) and the green fluorescent protein (GFP) from the same bicistronic mRNA transcript. Western blotting was used to assess the expression of HBsAg and of the endogenous gene GAPDH by using the following antibodies: Anti-Hepatitis B Virus Surface antigen (ABCAM) and anti-GAPDH antibody (Millipore) (Supplementary Figure S1).

Mutations associated with HCC were also introduced into a pIRES/ HBsAg plasmid according to the manufacturer instruction (QuikChange II XL Site-Directed Mutagenesis Kit, Agilent). HepG2 cells were transfected with pIRES-HBsAg plasmid encoding for wt and HBsAg mutants 24hours post cells plating by using the reagent Transit 2020 (MIRUS Bio). Cells were harvested after 7 days post transfection. Transfection efficiency was monitored by cotransfection of a vector expressing Secreted-Alkaline-Phosphatase (SEAP), which can be measured by colorimetric assay.

### Cellular cycle analysis

Transfected and harvested cells were permeabilized by using ethanol 70% in ice and then the DNA was stained with propidium iodide (PI)/RNAse A solution (Life Technologies). Cellular cycle analysis, including the evaluation of the percentage of cells in the G0/G1, S, and G2/M phases of the cell cycle, was performed by flow cytometry (BD FACS Calibur) only on transfected GFP+ cells, and analyzed by using the ModFIT and GraphPad softwares.

The production level of cyclin A (known to be crucial for cell cycle progression) was also evaluated by staining the permeabilized cells with the following antibody: CST 8607S; PE Mouse Anti-Human Cyclin A Set BD 550913.

## SUPPLEMENTARY MATERIALS FIGURES AND TABLES



## References

[1] Wang HC, Huang W, Lai MD, Su IJ Hepatitis B virus pre-S mutants, endoplasmic reticulum stress and hepatocarcinogenesis. Cancer Sci.

[2] Warner N, Locarnini S The antiviral drug selected hepatitis B virus rtA181T/sW172* mutant has a dominant negative secretion defect and alters the typical profile of viral rebound. Hepatology.

[3] El-Serag HB Hepatocellular carcinoma. N Engl J Med.

[4] Wen Y, Golubkov VS, Strongin AY, Jiang W, Reed JC Interaction of hepatitis B viral oncoprotein with cellular target HBXIP dysregulates centrosome dynamics and mitotic spindle formation. J Biol Chem.

[5] Pollicino T, Cacciola I, Saffioti F, Raimondo G Hepatitis B virus PreS/S gene variants: pathobiology and clinical implications. J Hepatol.

[6] Chen JJ, Tang YS, Huang SF, Ai JG, Wang HX, Zhang LP HBx protein-induced upregulation of microRNA-221 promotes aberrant proliferation in HBVrelated hepatocellular carcinoma by targeting estrogen receptor-alpha. Oncol Rep.

[7] Jiang SS, Huang SF, Huang MS, Chen YT, Jhong HJ, Chang IC, Chen YT, Chang JW, Chen WL, Lee WC, Chen MF, Yeh CT, Matsuura I Dysregulation of the TGFBI gene is involved in the oncogenic activity of the nonsense mutation of hepatitis B virus surface gene sW182*. Biochim Biophys Acta.

[8] Huang SF, Chen YT, Lee WC, Chang IC, Chiu YT, Chang Y, Tu HC, Yuh CH, Matsuura I, Shih LY, Lai MW, Wu HD, Chen MF Identification of transforming hepatitis B virus S gene nonsense mutations derived from freely replicative viruses in hepatocellular carcinoma. PLoS One.

[9] Neuveut C, Wei Y, Buendia MA Mechanisms of HBV-related hepatocarcinogenesis. J Hepatol.

[10] Sala M, Forner A, Varela M, Bruix J Prognostic prediction in patients with hepatocellular carcinoma. Semin Liver Dis.

[11] El-Serag HB Epidemiology of viral hepatitis and hepatocellular carcinoma. Gastroenterology.

[12] Liu CJ, Kao JH Hepatitis B virus-related hepatocellular carcinoma: epidemiology and pathogenic role of viral factors. J Chin Med Assoc.

[13] Li W, Goto K, Matsubara Y, Ito S, Muroyama R, Li Q, Kato N The characteristic changes in hepatitis B virus x region for hepatocellular carcinoma: a comprehensive analysis based on global data. PLoS One.

[14] Guo X, Jin Y, Qian G, Tu H Sequential accumulation of the mutations in core promoter of hepatitis B virus is associated with the development of hepatocellular carcinoma in Qidong, China. J Hepatol.

[15] Lee JH, Han KH, Lee JM, Park JH, Kim HS Impact of hepatitis B virus (HBV) x gene mutations on hepatocellular carcinoma development in chronic HBV infection. Clin Vaccine Immunol.

[16] Zhang X, Ding HG Key role of hepatitis B virus mutation in chronic hepatitis B development to hepatocellular carcinoma. World J Hepatol.

[17] Bruss V Hepatitis B virus morphogenesis. World J Gastroenterol.

[18] Yeh CT, Chen HC, Sung CM, Hsu CL, Lin CC, Pan KT, Tseng JH, Hung CF Retrospective comparison between a regular and a split-dose protocol of 5-fluorouracil, cisplatin, and mitoxantrone for the treatment of far advanced hepatocellular carcinoma. BMC Cancer.

[19] Lee SA, Kim K, Kim H, Kim BJ Nucleotide change of codon 182 in the surface gene of hepatitis B virus genotype C leading to truncated surface protein is associated with progression of liver diseases. J Hepatol.

[20] Stirk HJ, Thornton JM, Howard CR A topological model for hepatitis B surface antigen. Intervirology.

[21] Bruss V, Ganem D Mutational analysis of hepatitis B surface antigen particle assembly and secretion. J Virol.

[22] Schluter V, Rabe C, Meyer M, Koshy R, Caselmann WH Intracellular accumulation of middle hepatitis B surface protein activates gene transcription. Dig Dis.

[23] Salpini R, Alteri C, Cento V, Pollicita M, Micheli V, Gubertini G, De Sanctis GM, Visca M, Romano S, Sarrecchia C, Andreoni M, Angelico M, Parruti G Snapshot on drug-resistance rate and profiles in patients with chronic hepatitis B receiving nucleos(t)ide analogues in clinical practice. J Med Virol.

[24] Salpini R, Colagrossi L, Bellocchi MC, Surdo M, Becker C, Alteri C, Aragri M, Ricciardi A, Armenia D, Pollicita M, Di Santo F, Carioti L, Louzoun Y Hepatitis B surface antigen genetic elements critical for immune escape correlate with hepatitis B virus reactivation upon immunosuppression. Hepatology.

[25] Lai MW, Yeh CT The oncogenic potential of hepatitis B virus rtA181T/ surface truncation mutant. Antivir Ther.

[26] Bartholomeusz A, Locarnini S Hepatitis B virus mutants and fulminant hepatitis B: fitness plus phenotype. Hepatology.

[27] Chisari FV, Filippi P, Buras J, McLachlan A, Popper H, Pinkert CA, Palmiter RD, Brinster RL Structural and pathological effects of synthesis of hepatitis B virus large envelope polypeptide in transgenic mice. Proc Natl Acad Sci U S A.

[28] Bruss V Revisiting the cytopathic effect of hepatitis B virus infection. Hepatology.

[29] Huang SN, Chisari FV Strong, sustained hepatocellular proliferation precedes hepatocarcinogenesis in hepatitis B surface antigen transgenic mice. Hepatology.

[30] King KL, Cidlowski JA Cell cycle regulation and apoptosis. Annu Rev Physiol.

[31] Mun HS, Lee SA, Kim H, Hwang ES, Kook YH, Kim BJ Novel F141L pre-S2 mutation in hepatitis B virus increases the risk of hepatocellular carcinoma in patients with chronic genotype C infections. J Virol.

[32] Wang HC, Chang WT, Chang WW, Wu HC, Huang W, Lei HY, Lai MD, Fausto N, Su IJ Hepatitis B virus pre-S2 mutant upregulates cyclin A expression and induces nodular proliferation of hepatocytes. Hepatology.

[33] Aaltomaa S, Eskelinen M, Lipponen P Expression of cyclin A and D proteins in prostate cancer and their relation to clinopathological variables and patient survival. Prostate.

[34] Aaltomaa S, Lipponen P, Ala-Opas M, Eskelinen M, Syrjanen K, Kosma VM Expression of cyclins A and D and p21(waf1/cip1) proteins in renal cell cancer and their relation to clinicopathological variables and patient survival. Br J Cancer.

[35] Li JQ, Miki H, Wu F, Saoo K, Nishioka M, Ohmori M, Imaida K Cyclin A correlates with carcinogenesis and metastasis, and p27(kip1) correlates with lymphatic invasion, in colorectal neoplasms. Hum Pathol.

[36] Yang Y, Sun JW, Zhao LG, Bray F, Xiang YB Quantitative evaluation of hepatitis B virus mutations and hepatocellular carcinoma risk: a meta-analysis of prospective studies. Chin J Cancer Res.

