# Supplementary material for: Novel HBsAg mutations correlate with hepatocellular carcinoma, hamper HBsAg secretion and promote cell proliferation in vitro
Source: Oncotarget. 2017 Feb 1;8(9):15704–15. doi: 10.18632/oncotarget.14944 (PMC5362517; doi:10.18632/oncotarget.14944)
Supplement: Supplementary file 1 [file oncotarget-08-15704-s001.pdf]

Novel HBsAg mutations correlate with hepatocellular carcinoma, hamper HBsAg secretion and promote cell proliferation *in vitro*

SUPPLEMENTARY TEXT I

Protocol for pre-S1, pre-S2 and S-HBsAg region sequencing

HBV-DNA was extracted using a commercially available kit (QIAmp DNA blood mini-kit, Qiagen Inc., USA), and then amplified with Amplitaq-Gold polymerase using the following primer pairs: F1-5'GGTCACCATATTCTTGGGAA and R1-5'GTGGGGGTTGCGTCAGCAAA. Polimerase chain reaction (PCR) conditions were: one cycle at 93 °C for 12 min, 40 cycles (94 °C 50 s, 57 °C 50 s, 72 °C 1 min and 30 s), and a final cycle at 72 °C for 10 min. For samples with serum low HBV-DNA, 2 additional heminested-PCR were performed, starting from the same first amplicon: eminested_1 used the following primer pairs (F1-5'GGTCACCATATTCTTGGGAA and R2-GAGGACAAACGGGCAACATACCTT and eminested-2 used F2-GTTGACAAGAATCCTCACAATA and R1-5'GTGGGGGTTGCGTCAGCAAA. Both eminested-PCRs conditions were: one cycle at 93 °C for 12 min, 35 cycles (94 °C 50 s, 56 °C 50 s, 72 °C 1 min), and a final cycle at 72 °C for 10 min. PCR-products were purified and sequenced by using eight different overlapping sequence-specific primers, a BigDye terminator v. 3.1 cycle sequencing kit (Applied-Biosystems FosterCity, CA) and an automated sequencer (Genetic Analyzer 3130XL). The sequences were analyzed using SeqScape-v.2.5 software. The quality endpoint for each individual gene was ensured by a coverage of the S gene sequence by at least two segments. Sequences having a mixture of wild-type and mutant residues at single positions were considered to have the mutant(s) at that position.

SUPPLEMENTARY TEXT II

Protocol for HBsAg ultra-deep pyrosequencing (UDPS)

PCR-barcoded primers were designed to amplify four partially overlapping amplicons covering the full-length reverse transcriptase region, and the overlapping complete region of the S-HBsAg. In particular, four fragments of 424, 332, 384 and 427 nucleotides, encompassing the Reverse Transcriptase (RT) and S genomic region, were generated by nested PCR using barcoded-modified primers after a first amplification. Nested-PCR was performed with the Fast Start HiFi PCR system (Roche Diagnostics, Mannheim, Germany) under the following conditions: 1 cycle of 94°C for 3 min, 30 cycles of 94°C for 30 s, 60°C for 30 s, 72°C for 35sec, followed by a final extention at 72°C for 7 min. The amplified products were purified using Agencourt AMPure XP PCR purification beads (Beckman Coulter, Brea, CA) and quantified with the Quant-iT PicoGreen dsDNA Assay Kit (Life Technologies, Eugene, Oregon USA) by GloMax Multidetection System (Promega, Wisconsin USA). Equimolar pooled PCR products were clonally amplified on captured beads in water-oil emulsion microreactors (1.2 DNA-copies/beads). A total of 500,000 DNA-enriched beads were deposited in the GS Junior Titanium PicoTiterPlate device and pyrosequenced in both forward and reverse directions. The 200 nucleotide cycles were performed in a 10h sequencing run.

The HBV- S-HBsAg region sequences obtained after 454-pyrosequencing were de-multiplexed and quantified using the standard flowgram format (SFF) tool by Roche. By an home-made pearl script calling for Blast algorithm, all reads were checked for homology with reference strains (> 80%) and the orientation was discerned [1]. After this filtering, SHORAH package 0.5.1 [2] were used to correct sequences for homolymeric region associated errors, using as reference the sequences obtained from population sequencing. Subsequently the sequences were clustered, taking into account for reads redundancy and forward/reverse directions, using Exonerate 2.2.0 package [3] and aligned against the HBV consensus D (GenBank accession number: X65259) using Needleman–Wunsch algorithm (Emboss package) [4, 5]. The final alignments were manually checked for insertion or deletion in homopolymeric region that would results in a frame shift. At each position the coverage and the amino acid variants were evaluated by a home-made pearl script. Phylogenetic analyses were performed to avoid any possible contamination or mixing among the pooled samples analyzed.

Sensitivity of UDPS for detecting minor HBV-RT/S-HBsAg variants

The frequency of errors resulting from RT/S-HBsAg amplification and deep sequencing was assessed using a plasmid containing the S-HBsAg gene as control. Mismatch nucleotide error rate was very low after sequencing correction (<0.006%). Furthermore, considering only the variants covered in both orientations

in more than 5 reads, we never found mismatch in the control plasmid, thus only variants with these characteristic were considered reliable for the analyses.

SUPPLEMENTARY TEXT III

The following primers were used for site-directed mutagenesis:

P203Q_Forward (F): 5'GTGGTATTGGGGGCAAAGTCTGTACAGCATC3';

P203Q_Reverse (R): 3'GATGCTGTACAGACTTTGCCCCCAATACCAC5';

S210R_F: 5'CTGTACAGCATCTTGAGGCCCTTTTTACCGC3';

S210R_R: 3'GCGGTAAAAAGGGCCTCAAGATGCTGTACAG5';

P203Q+S210R_F: 5'GTATTGGGGGCAAAGTCTGTACAGCATCTTGAGGCCCTTTTTAC3';

P203Q+S210R_R: 3'GTAAAAAGGGCCTCAAGATGCTGTACAGACTTTGCCCCCAATAC5'.

REFERENCES

1. Altschul SF, Gish W, Miller W, Myers EW, Lipman DJ. Basic local alignment search tool. J Mol Biol. 1990;215:403-410.
2. Zagordi O, Bhattacharya A, Eriksson N, Beerenwinkel N. ShoRAH: estimating the genetic diversity of a mixed sample from next-generation sequencing data. BMC Bioinformatics. 2011;12:119.
3. Slater GS, Birney E. Automated generation of heuristics for biological sequence comparison. BMC Bioinformatics. 2005;6:31.
4. Needleman SB, Wunsch CD. A general method applicable to the search for similarities in the amino acid sequence of two proteins. J Mol Biol. 1970;48:443-453.
5. Rice P, Longden I, Bleasby A. EMBOSS: the European Molecular Biology Open Software Suite. Trends Genet. 2000;16:276-277.

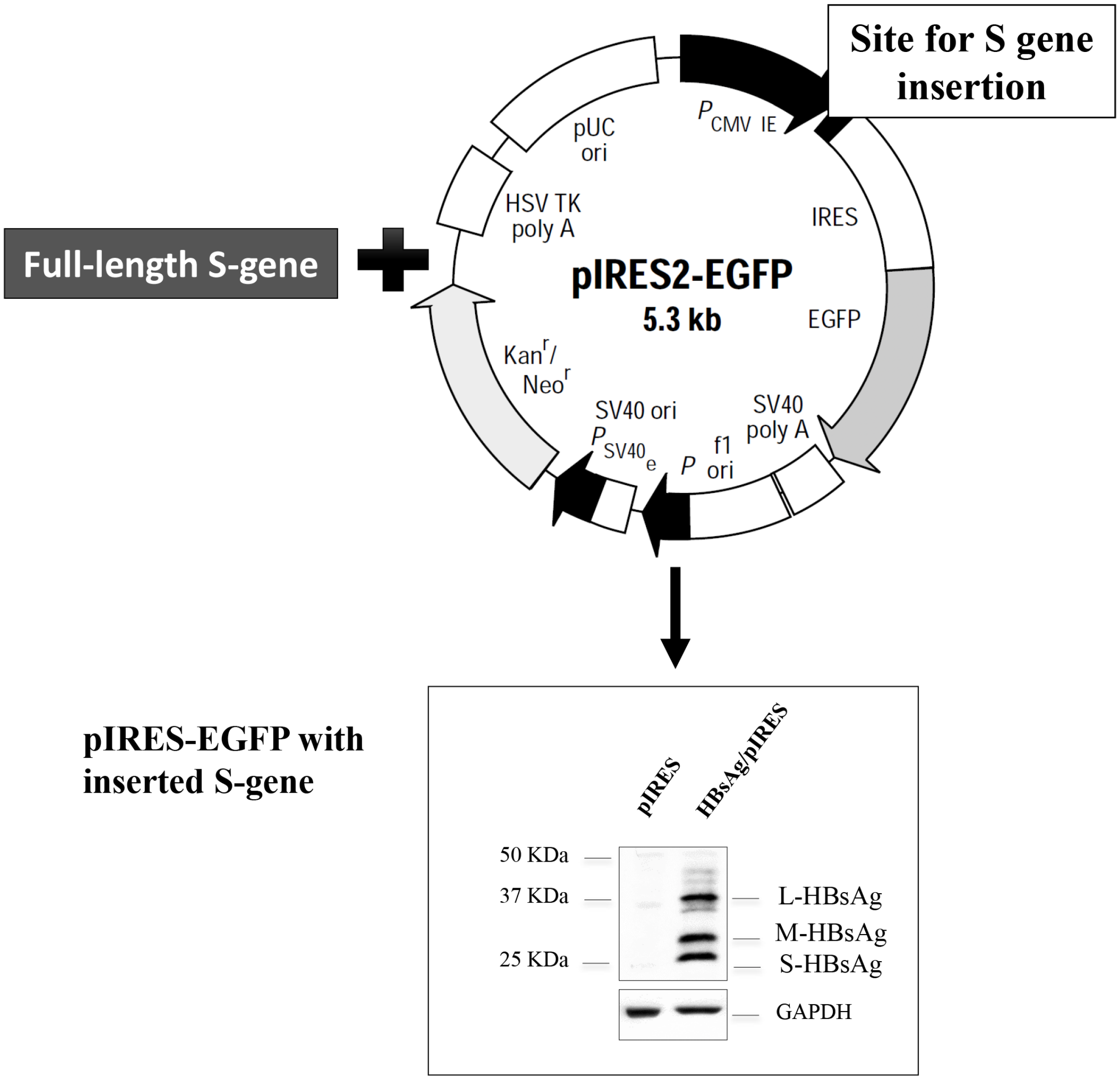

Supplementary Figure 1: Cloning strategy for the insertion of full-length HBsAg sequence into the pIRES2-EGFP plasmid and confirmation of wild type HBsAg expression by using Western Blot.

Supplementary Table 1: Characteristics of patients with UDPS results available

Characteristics	Pyro HCC population (N=13)	Bulk HCC population (N=23)	P value	Pyro non HCC population (N=27)	Bulk non HCC population (N=105)	P value
Male, N(%)	11 (84.6)	21 (91.7)	0.6	17 (63.0)	80 (76.2)	0.2
Italian Nationality, N(%)	8 (61.5)	16 (69.6)	0.7	18 (70.4)	89 (84.8)	0.05
Median Age [Years] (IQR)	66 (54-70)	63 (53-70)	0.9	52 (43-63)	51 (41-65)	1.0
Median ALT [IU/L] (IQR)	103 (59-112)	82 (28-112)	0.6	34 (25-75)	49 (29-90)	0.3
Median AST [IU/L] (IQR)	61 (40-112)	50 (33-139)	0.7	31 (24-73)	35 (23-58)	0.8
Median HBV-DNA [log IU/ml]	5.0 (2.7-6.4)	4.0 (2.1-6.5)	0.6	3.8 (2.6-6.5)	4.1 (3.1-5.4)	0.9
Gen A	6 (46.2)	6 (26.1)	0.3	3 (11.1)	29 (27.6)	0.1
Gen D	7 (53.8)	17 (73.9)		24 (88.9)	76 (72.4)	
P203Q	3 (23.1)	4 (17.4)	0.7	0 (0)	1 (1.0)	1.0
S210R	6 (46.2)	8 (34.8)	0.7	2 (7.4)	4 (3.8)	0.6
P203Q+S210R	3 (23.1)	4 (17.4)	0.7	0 (0)	0 (0)	1.0
